# Chameleonic Chloroma: A Case of Myeloid Sarcoma Presenting as a Pancreatic Head Mass

**DOI:** 10.7759/cureus.57880

**Published:** 2024-04-08

**Authors:** Benjamin F Cardenas, Ayeetin M Azah, Azfar S Syed, Jeffrey T Laczek

**Affiliations:** 1 Internal Medicine, Walter Reed National Military Medical Center, Bethesda, USA; 2 Gastroenterology, Walter Reed National Military Medical Center, Bethesda, USA

**Keywords:** granulocytic sarcoma, acute myeloid leukemia (aml), chloroma, pancreatic cancer, myeloid sarcoma

## Abstract

We report a case of pancreatic myeloid sarcoma (MS), an extremely rare manifestation of acute myeloid leukemia (AML), in a 35-year-old male who presented with epigastric pain and watery stools. Initial diagnostic testing was inconclusive; however, following an extensive evaluation, endoscopic biopsies suggested AML, which was confirmed by a bone marrow biopsy. Given that few cases are documented in the literature, pancreatic MS without a preexisting hematologic malignancy poses a significant diagnostic challenge.

## Introduction

Acute myeloid leukemia (AML) can present with extramedullary mass lesions known as myeloid sarcomas (MSs), or chloromas, in less than 1% of cases [1]. MS has been observed in a wide variety of tissue sites and can mimic alternative primary cancers, leading to delayed diagnoses and inappropriate treatments [2]. Pancreatic MS is a poorly understood and rarely documented condition, with few cases reported in the literature involving patients who presented without a preexisting hematological malignancy. This report presents, and discusses the challenges of, a case of pancreatic head myeloid sarcoma masquerading as pancreatic ductal adenocarcinoma.

## Case presentation

**Figure 1:**
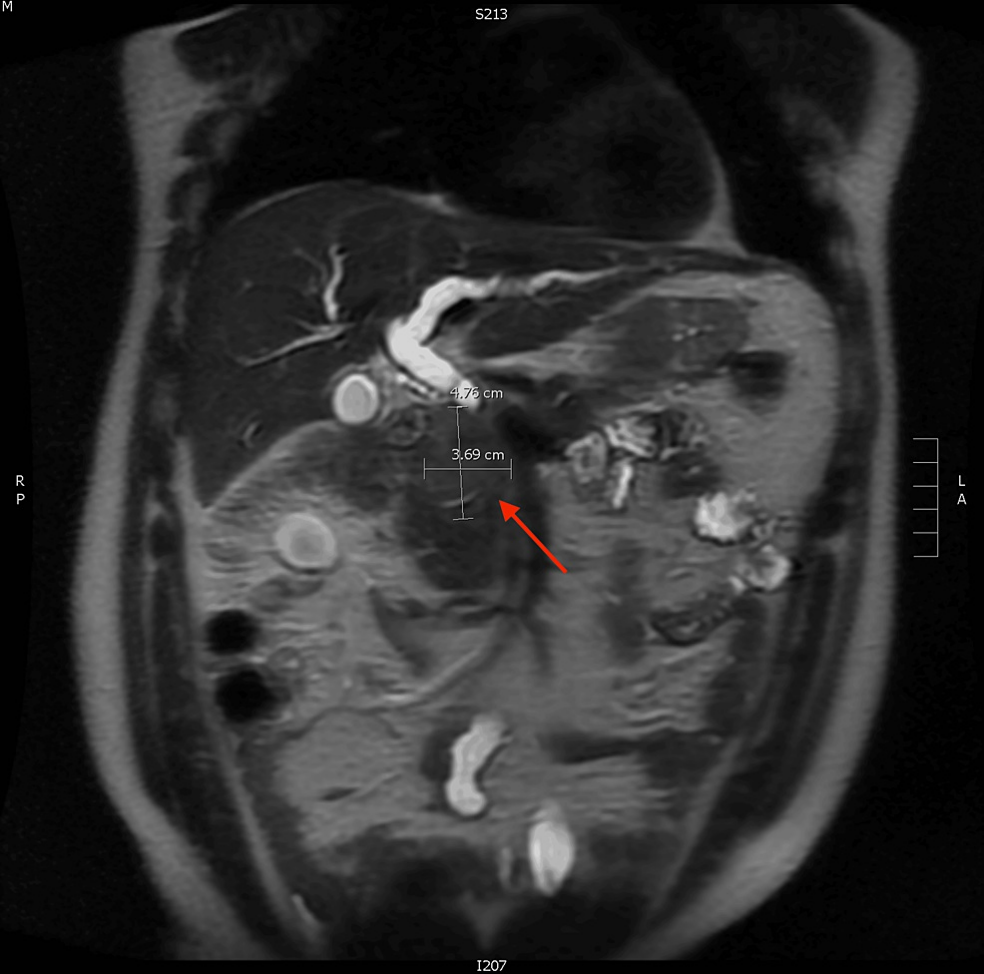
Magnetic resonance cholangiopancreatography (coronal view) demonstrating a 4.8 x 4.0 x 3.7 cm homogeneous hypo-enhancing mass (red arrow) in the pancreatic head region and common bile duct dilation.

**Figure 2:**
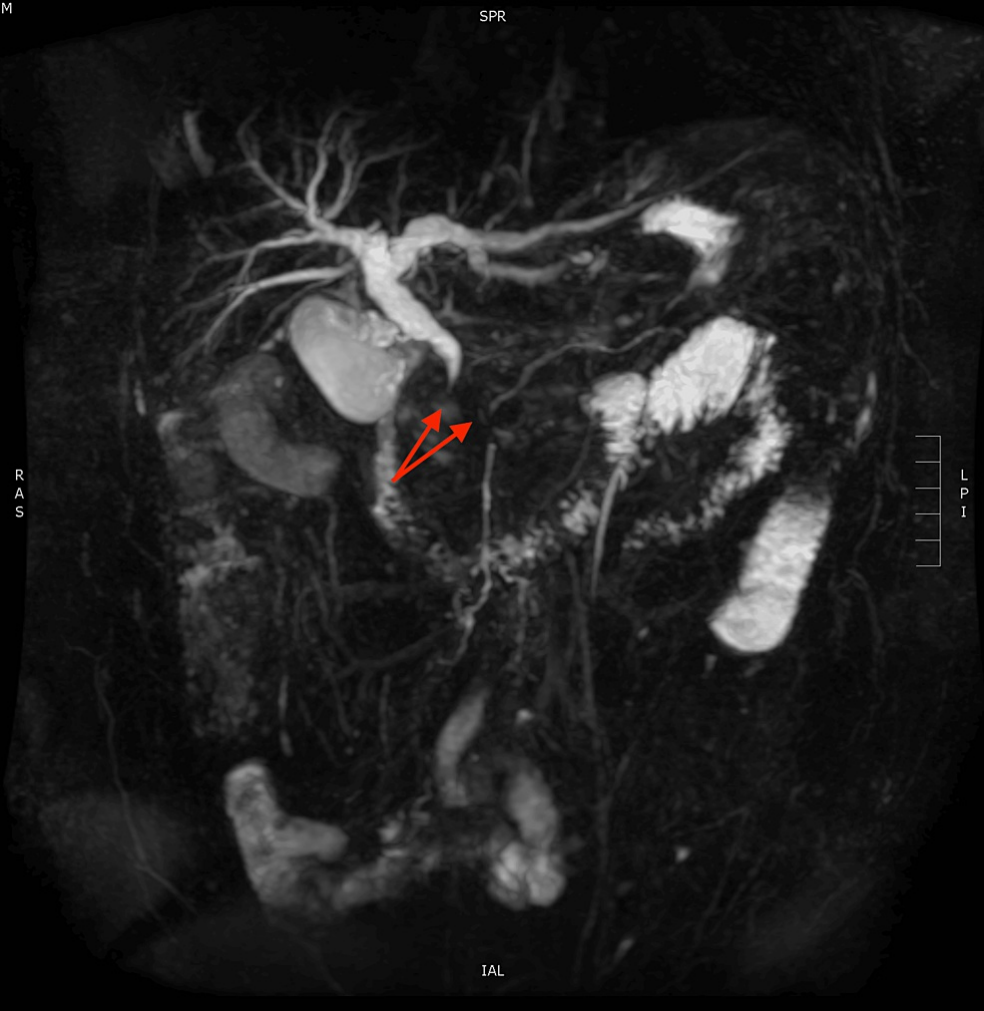
3D reconstruction of the pancreaticobiliary system demonstrating abrupt cessation of the common bile duct and pancreatic duct, or double duct sign (red arrows).

A previously healthy 35-year-old man presented to the emergency department with one week of worsening epigastric pain radiating to his back. He also reported bloating and watery, pale-colored stools. On examination, he had visible jaundice and laboratory tests revealed conjugated hyperbilirubinemia (direct bilirubin 3.2 mg/dL (reference <0.3 mg/dL)) and significant elevations in liver-associated enzymes (ALT 548 (<33 U/L), AST 332 (<20 U/L)). Additionally, he had mild leukopenia (WBC 3.5 x 10^9^/L (4-10 x 10^9^/L)) and mild anemia (Hgb 13.0 g/dL (13.8-17.2 g/dL)). An abdominal ultrasound demonstrated dilation of the common bile duct (10 mm). Magnetic resonance cholangiopancreatography (MRCP) revealed a 4.8 x 4.0 x 3.7 cm homogeneous hypo-enhancing mass in the pancreatic head with a double duct sign (Figures 1, 2), raising concerns for pancreatic ductal adenocarcinoma (PDAC). He was admitted for an expedited work-up of the mass and management of biliary obstruction.

Endoscopic ultrasound (EUS) showed a pancreatic head mass with fine needle aspiration (FNA) showing lymphocytes. Endoscopic retrograde cholangiopancreatography (ERCP) showed a stricture in the distal common bile duct, and a biliary stent was placed. Repeat EUS/FNA was pursued two weeks later, showing a large hypoechoic lymph node in the region of the pancreatic head; FNA again showed lymphocytes.

**Figure 3:**
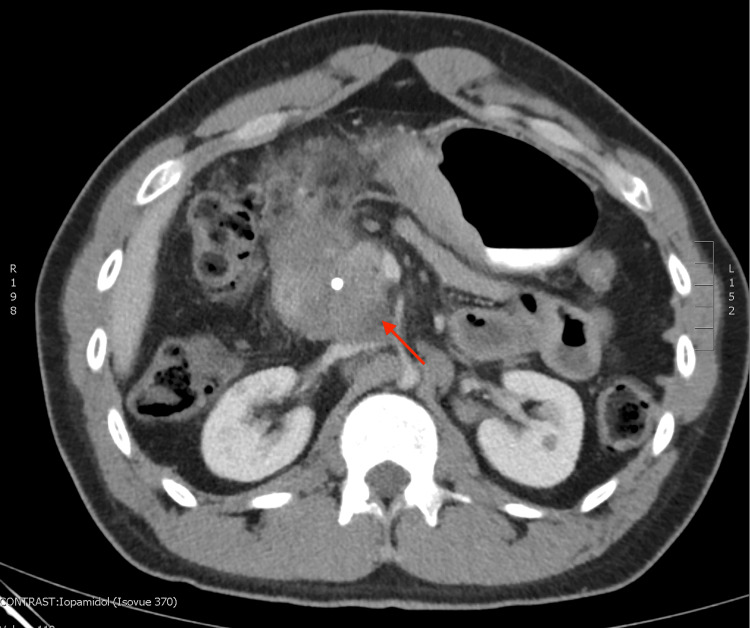
Abdominal computed tomography (axial view) with oral and IV contrast re-demonstrating homogeneous mass, 5.6 cm in the longest dimension, in the region of the pancreatic head circumferentially surrounding the common bile duct stent.

**Figure 4:**
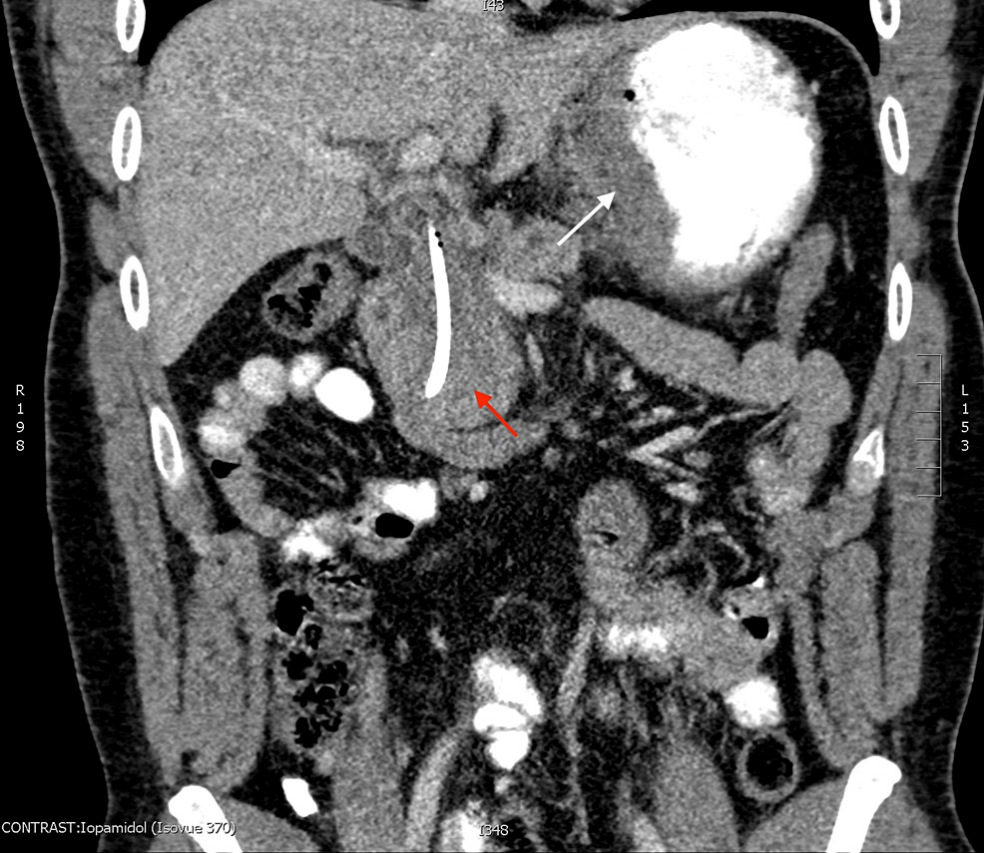
Contrast artifact versus food products versus later discovered gastric mass can be seen in the lesser gastric curvature (white arrow); notably, this was not commented on in the original radiography report. The red arrow shows the pancreatic head mass.

An abdominal CT scan was performed to further evaluate his pancreatic head lesion and demonstrated a 5.6 cm ill-defined homogenous hypodense pancreatic head mass (Figures 3, 4) with new omental caking and retroperitoneal lymphadenopathy. At this point in time, the most likely diagnosis was believed to be PDAC, and the patient was not considered a surgical candidate due to superior mesenteric artery encasement and the concern for metastatic disease.

**Figure 5:**
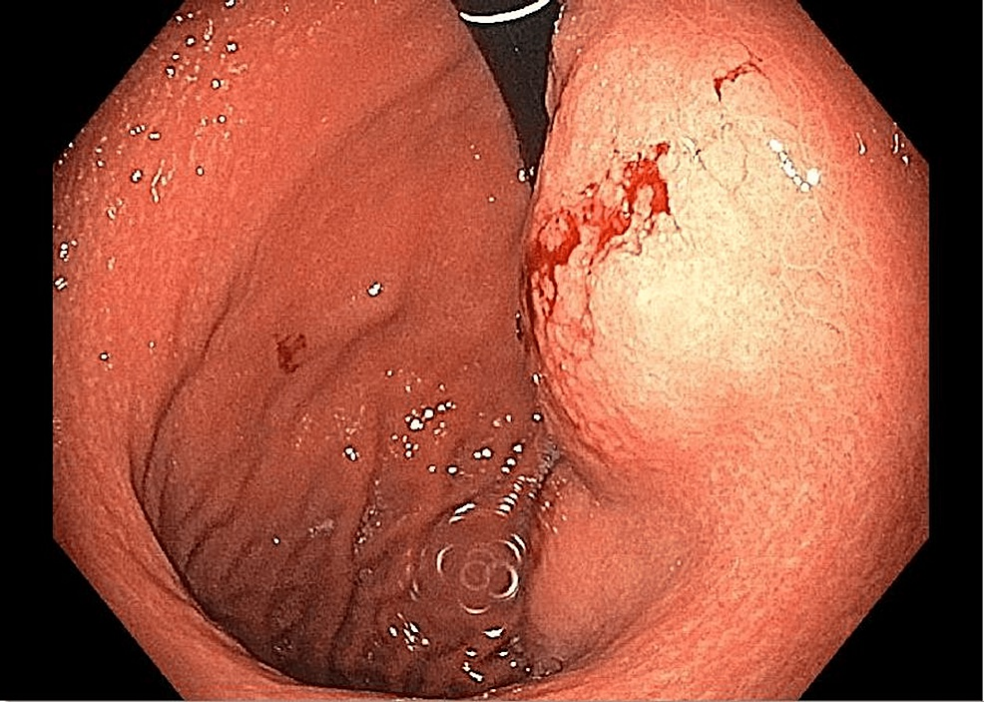
Endoscopic image of the retrograde view of a large, smooth mass along the lesser gastric curvature. Gross bleeding is present following an excisional biopsy of the mass.

**Figure 6:**
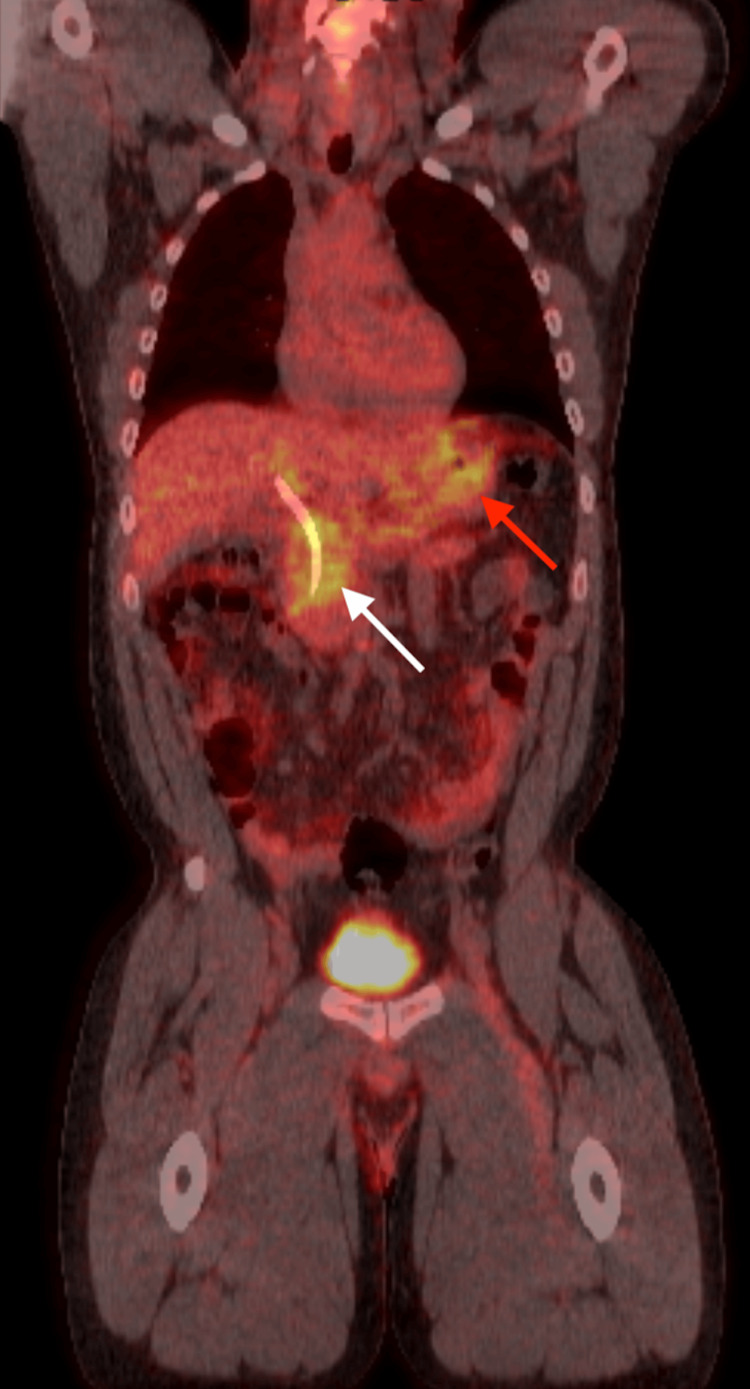
Positron emission tomography (coronal view) demonstrating abnormally elevated F-FDG uptake in the pancreatic head (white arrow) and gastrum (red arrow).

In search of further biopsy sites, a repeat endoscopy was performed, showing what appeared to be a subepithelial mass on the distal lesser curvature of the stomach (Figure 5). A biopsy of this mass demonstrated a high-grade myeloid neoplasm. Bone marrow biopsy was then performed, showing 16% blasts (<5%), solidifying a diagnosis of AML and MS. Genotyping demonstrated intermediate-risk FLT-3 positive disease. Positron emission tomography (PET) scan demonstrated hypermetabolic pancreatic, gastric, retroperitoneal, tonsillar, and cervical lymph nodes (Figure 6).

The patient completed 7+3 induction chemotherapy with daunorubicin, cytarabine, and midostaurin with a morphologic complete response. He has since completed consolidation chemotherapy with cytarabine and gilteritinib. He remains in remission and is undergoing surveillance without active plans for allogeneic hematopoietic stem cell transplant (HCT).

## Discussion

Pancreatic MS is a dangerous mimicker of PDAC. When misdiagnosed, this can lead to inappropriate treatment, including major surgeries such as the Whipple procedure [3-5]. This can also lead to delay in appropriate treatment, leading to death [6]. Myeloid sarcoma has a poor prognosis with a median survival of four months from the time of diagnosis. This is in part due to delayed diagnosis and in part due to the inherent aggressiveness of the disease [2], necessitating expeditious diagnosis and appropriate treatment that is distinctly different from traditional treatment for PDAC.

Diagnosis can be difficult when MS presents as a pancreatic mass. Presenting symptoms are identical to PDAC when a biliary obstruction is present [7]. Early ERCP is necessary depending on the extent of obstruction, and EUS/FNA is often an early utilized diagnostic modality. In pancreatic MS, EUS/FNA may demonstrate lymphoid cells [8], but the cytology from an FNA is generally insufficient to make a diagnosis. Of note, multiple cases demonstrated inconclusive biopsies despite satisfactory visualization on EUS [5,6].

Imaging modalities, such as PET, CT, and MRI, are an integral part of detecting pancreatic MS as they can reveal more easily accessible and less invasive sites for biopsy than the pancreas itself. The presence of distant lesions that would be less common for pancreatic carcinoma metastasis, such as orbital involvement, can be helpful in some cases; however, myeloid sarcoma can also exist as a solitary mass with or without local spread [1]. Data in the literature on differentiating a chloroma from pancreatic adenocarcinoma based on imaging characteristics of the mass alone are insufficient. Both lesions can be hypodense on CT and can show enhancement on F-FDG PET [9,10].

Gastroenterologists should be willing to pursue short-interval repeat diagnostic testing when the initial work-up is inconclusive. Both MS and pancreatic carcinoma can be aggressive with rapid development of metastatic spread. After only two weeks, new sites of involvement were present in our case, prompting the search for a new biopsy site and leading to a diagnosis and subsequent treatment. A further delay in diagnosis would have been detrimental to our patient’s overall outcome.

When a diagnosis of myeloid sarcoma is established or suspected, early consultation with oncological specialists is imperative to guide further diagnostic/staging procedures and treatment. The management of myeloid sarcoma is not well established but is generally agreed to follow treatment pathways similar to AML without MS, including induction chemotherapy with or without consolidation HCT [11]. Overall survival at three years with HCT may be as high as 46%, and 39-47% at five years [12], significantly higher than the overall prognosis with MS. Even with solitary MS without bone marrow involvement, systemic therapy is the mainstay of treatment, and surgery plays a primarily palliative role [11].

## Conclusions

MS is a rare disease that can present in almost any organ system and is often initially misdiagnosed as alternative primary malignancies. Pancreatic MS presents an especially difficult diagnostic challenge for practitioners who are not familiar with the disease as it can present similarly to pancreatic adenocarcinoma, a much more common and similarly dangerous malignancy. The case presented in this report demonstrates an individual who presented with this rare mimic and benefited from repeat diagnostic testing to secure the correct diagnosis and begin appropriate life-saving treatment.
